# Tuberous Sclerosis Complex and the kidneys: what nephrologists need to know

**DOI:** 10.1590/2175-8239-JBN-2024-0013en

**Published:** 2024-07-05

**Authors:** Aline Grosskopf Monich, John J. Bissler, Fellype Carvalho Barreto

**Affiliations:** 1 Universidade Federal do Paraná, Departamento de Clínica Médica, Programa de Pós-Graduação em Medicina Interna e Ciências da Saúde, Curitiba, PR, Brazil.; 2 Hospital Universitário Evangélico Mackenzie, Serviço de Nefrologia, Curitiba, PR, Brazil.; 3 University of Tennessee, Health Science Center, Le Bonheur Children's Hospital, Department of Pediatrics, Memphis, TN, USA.; 4 Le Bonheur Children's Hospital, Children's Foundation Research Institute, Memphis, TN, USA.; 5 St. Jude Children’s Research Hospital, Pediatric Medicine Department, Memphis, TN, USA.; 6 Universidade Federal do Paraná, Departamento de Clínica Médica, Serviço de Nefrologia, Curitiba, PR, Brazil.

**Keywords:** Tuberous Sclerosis, Angiomyolipoma, MTOR Inhibitors, Renal Insufficiency, Chronic

## Abstract

Tuberous sclerosis complex (TSC) is an autosomal dominant disease characterized by the development of hamartomas in the central nervous system, heart, skin, lungs, and kidneys and other manifestations including seizures, cortical tubers, radial migration lines, autism and cognitive disability. The disease is associated with pathogenic variants in the *TSC1* or *TSC2* genes, resulting in the hyperactivation of the mTOR pathway, a key regulator of cell growth and metabolism. Consequently, the hyperactivation of the mTOR pathway leads to abnormal tissue proliferation and the development of solid tumors. Kidney involvement in TSC is characterized by the development of cystic lesions, renal cell carcinoma and renal angiomyolipomas, which may progress and cause pain, bleeding, and loss of kidney function. Over the past years, there has been a notable shift in the therapeutic approach to TSC, particularly in addressing renal manifestations. mTOR inhibitors have emerged as the primary therapeutic option, whereas surgical interventions like nephrectomy and embolization being reserved primarily for complications unresponsive to clinical treatment, such as severe renal hemorrhage. This review focuses on the main clinical characteristics of TSC, the mechanisms underlying kidney involvement, the recent advances in therapy for kidney lesions, and the future perspectives.

## Introduction

The first reports of the disease currently recognized as tuberous sclerosis complex (TSC), trace back to 1835 through illustrations by Pierre François Olive Rayer, showcasing papular lesions on a man’s face^1^. Subsequently, in 1862, Friedrich Daniel von Recklinghausen presented a case to the Berlin Obstetrics Society involving a newborn with multiple heart tumors and cerebral sclerosis^1^. However, it was Désiré-Magloire Bourneville who, in 1880, provided the first detailed description of the central nervous system involvement, officially naming the condition tuberous sclerosis^1,2^.

TSC is an autosomal dominant disorder marked by the formation of hamartomas in various organs, including the kidneys, brain, lungs, heart, and skin. Additionally, other neurological findings and symptoms, such as cortical tubers, radial migration lines, seizures, cognitive impairment, and autism, may be present^3–6^. The incidence of TSC ranges from 1/6,000 to 1/10,000 live births, with an estimated 2,000,000 individuals worldwide currently affected by the disease^4^. Although TSC prevalence is consistent across populations, ethnicities, and genders, certain manifestations, such as pulmonary lymphangioleiomyomatosis (LAM) and renal angiomyolipoma (AML), appear to be more pronounced in females, suggesting hormonal influence^7^. Renal involvement may develop from childhood. Overtime, renal lesion progression, mainly angiomyolipoma, ultimately leads to renal hemorrhage, emergency surgical interventions, and decreased renal function^3,6^. The introduction of mTOR inhibitors (mTOR) in the late 2000s has changed the natural history of TSC, significantly enhancing the quality of life and survival rates for affected individuals^4,6^.

Despite recent advances on molecular diagnosis and the development targeted therapies, patients with rare diseases such as TSC are still neglected^8^. In Brazil, epidemiological data on TSC are scarce. Initial findings from an observational study on renal involvement in TSC revealed that only 25% of patients were using mTOR inhibitors, and partial or total nephrectomy are still routinely performed in these patients^9^. A comprehensive understanding of TSC is imperative for ensuring early diagnosis and appropriate medical follow-up and treatment. This review encompasses genetic aspects, clinical characteristics of the disease with a focus on renal manifestations and addresses key aspects of diagnosis and treatment of renal lesions associated with TSC.

## Genetic Aspects

**Figure 1:**
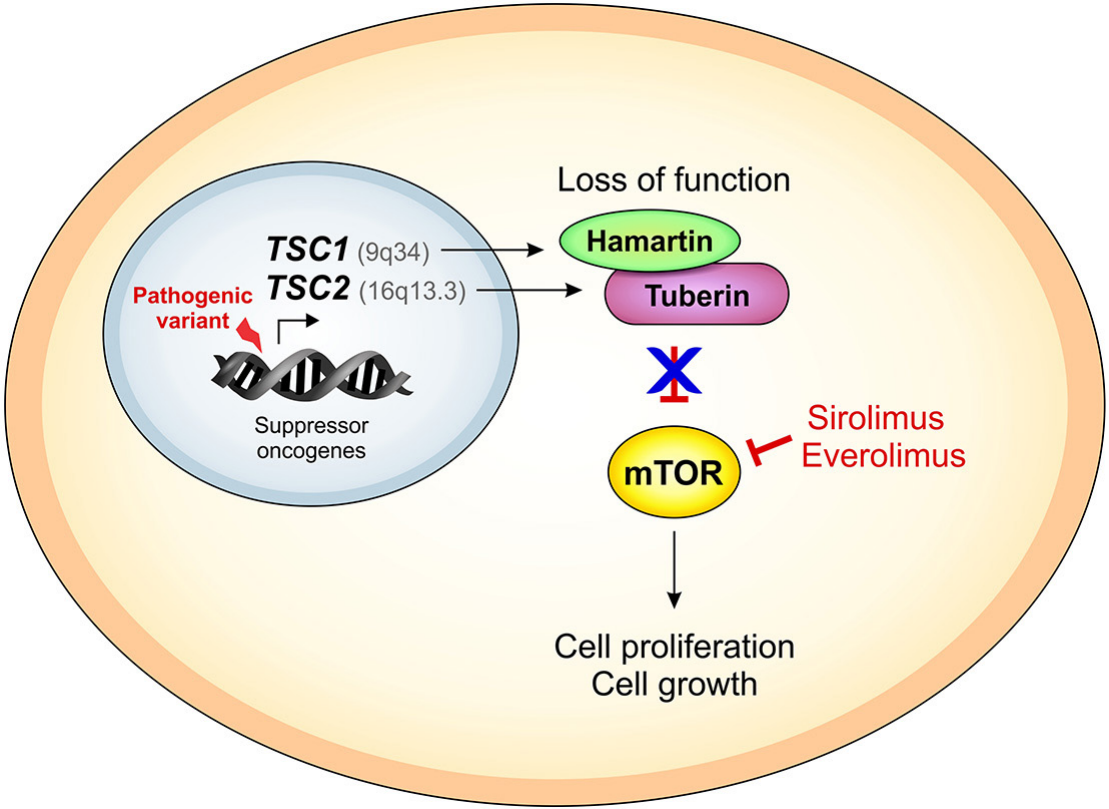
Dysfunction of the mTOR pathway and mechanism of action of mTOR inhibitors in tuberous sclerosis complex.

TSC is caused by pathogenic variants in the *TSC1* and *TSC2* genes*,* recognized as tumor suppressor genes, located on chromosomes 9q34.13 and 16p13.31, respectively^10^. These genes are co-expressed in all nucleated cells and thus in multiple organs, such as the lungs, kidneys, brain, and pancreas. In healthy individuals, the gene products tuberin and hamartin form heterodimers with high affinity, forming a complex that negatively regulates the *mechanistic target of rapamycin (mTOR)* cascade. This cascade plays a pivotal role in cell growth and proliferation through ribosomal biosynthesis and protein synthesis^10^. Patients with TSC have pathogenic variants in either *TSC1* or *TSC2*, not both genes; these pathogenic variants functionally inactivate *TSC1* or *TSC2* or leads to the loss of the ideal conformation of the hamartin-tuberin complex causing aberrant activation of mTOR and resulting in heightened cell proliferation and growth (Figure 1)^4,10,11^. While the loss of a single allele of *TSC1* or *TSC2* may be sufficient to induce certain clinical features of TSC, such as neuropsychiatric changes, the development of hamartomas appears to require an additional somatic inhibitory mutation, termed a second hit, in the remaining allele. This aligns with Knudson’s classic two-hit hypothesis^4,10,12^. Second hits are often identified in the remaining allele of *TSC1* or *TSC2* in most TSC-related AML and renal cell carcinomas^13,14^.

A diverse array of pathogenic variants exists in both genes. Pathogenic variants in *TSC1* are often small insertions or deletions that result in shortened protein. Pathogenic variants in *TSC2* include large deletions, nonsense mutations (when a point mutation in a sequence of DNA results in a premature stop codon that cause cessation of translation and prevent the synthesis of a complete protein), and missense mutations (when a single nucleotide change results in a different aminoacid)^10^. According to the *Leiden Open Variation Database,* over 900 pathogenic allelic variants in *TSC1* and 2700 in *TSC2* have been reported to date^15^. Notably, pathogenic variants in *TSC2* are more prevalent than in *TSC1*, with missense pathogenic variants and significant genomic deletions being more commonly observed in TSC2^10^. *De novo* pathogenic variants constitute approximately 80% of TSC cases, being approximately four times more common in *TSC2* than in *TSC1*. In familial cases, there is no difference between the prevalence of pathogenic variants in *TSC1* and *TSC2* ^10,13^. Despite the absence of a clear genotype-phenotype correlation, pathogenic variants in *TSC2* are often associated with more severe clinical manifestations of renal angiomyolipoma, cognitive impairment, and epilepsy^10^.

## Clinical Manifestations and Diagnostic Criteria

There exists a broad spectrum of phenotypes in terms of age of onset, clinical manifestations, severity, and number of lesions^5,16^. Approximately 90% of patients exhibit skin lesions, 90% present with some neurological sign or symptom, and 75–80% display renal abnormalities^16^.

**Figure 2:**
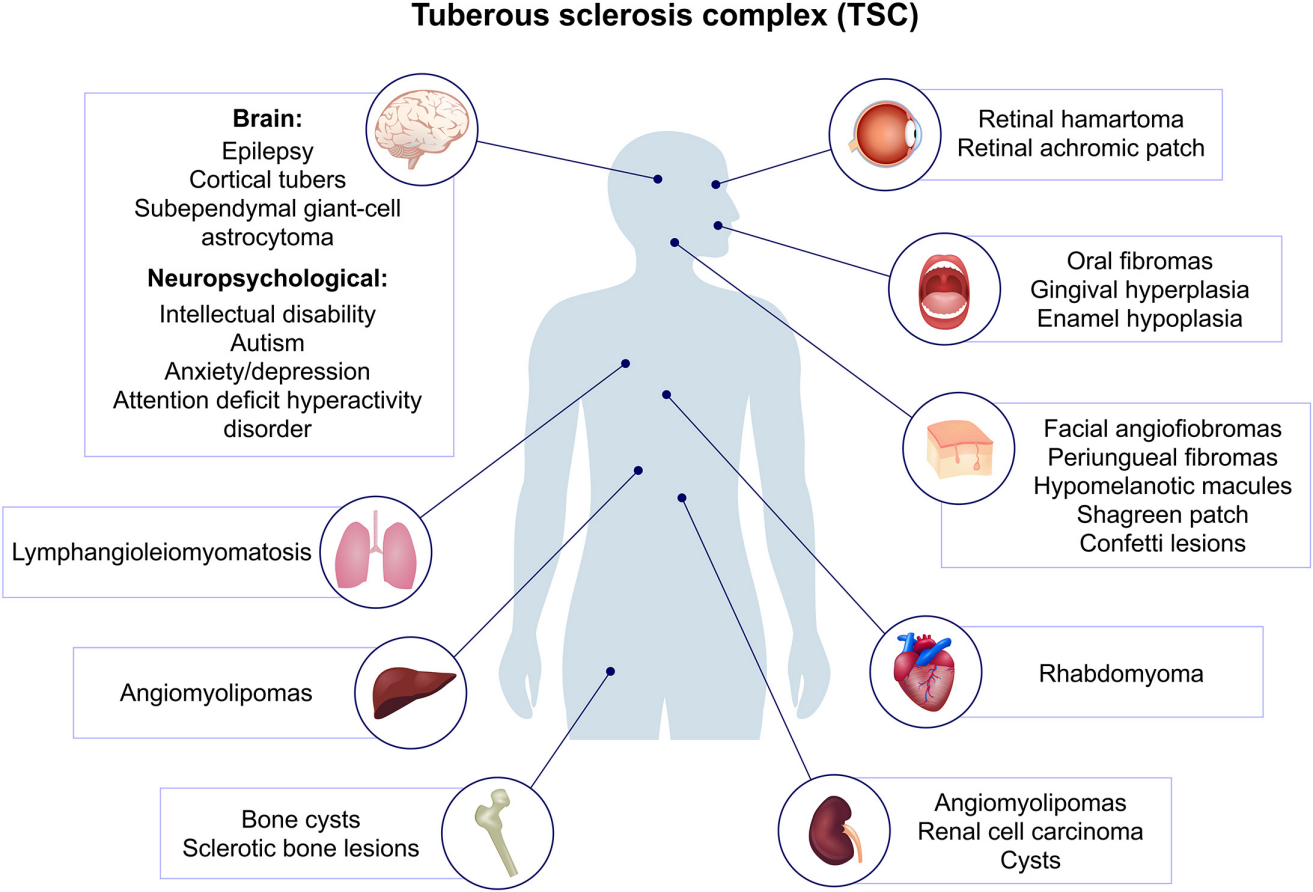
Clinical manifestations of Tuberous Sclerosis Complex.

Clinical manifestations have distinctive characteristics in terms of symptom onset. Cardiac rhabdomyomas can be detected during intrauterine life in 90% of affected individuals and often regress in early childhood. Infants frequently develop a specific form of epilepsy characterized by spasms, subependymal giant cell astrocytomas (SEGA), epilepsy, cognitive difficulties, and neuropsychiatric disorders, collectively termed TSC-associated neuropsychiatric disorders (TAND), which are recognizable in early infancy. Dermatological manifestations are typically diagnosed in childhood, renal manifestations may begin during childhood and persist into adulthood, while LAM primarily occurs in women from adolescence onwards, potentially linked to hormonal factors^5,7,16^ (Figure 2).

The diagnosis of TSC can be established through genotyping and/or clinical manifestations^17^. The identification of a pathogenic variant in *TSC1* or *TSC2* is sufficient for the diagnosis of TSC, irrespective of clinical findings, as *TSC* manifestations may emerge at different ages^17^. However, in 10 to 15% of TSC patients meeting clinical diagnostic criteria, the pathogenic variant may elude identification through conventional genetic tests. This could be attributed to the presence of mosaicism and intronic mutations, indicating that the absence of an identified pathogenic variant does not preclude the diagnosis of TSC^14^.

The clinical criteria for diagnosing TSC have recently been revised^17^. A definitive clinical diagnosis requires the presence of two major criteria or one major and two minor criteria. The presence of one major or two minor criteria suggests a possible diagnosis of TSC (Chart 1)^17^.

**Chart 1:** Diagnostic criteria for tuberous sclerosis complex (TSC)

**Genetic criteria** * Presence of a pathogenic variant in *TSC1* or *TSC2*	
**Clinical criteria** Two major criteria or one major and two minor criteria required	
**Major criteria**	**Minor criteria**
Angiofibromas (≥ 3) or cephalic fibrous plaque	Dental enamel pits (≥ 3)
Ungueal fibromas (≥ 2)	Intraoral fibromas (≥ 2)
Hypomelanotic macules (≥ 3, at least 5 mm in diameter)	Nonrenal hamartoma
Shagreen patch	Retinal achromic patch
Multiple retinal hamartomas	Multiple renal cysts
Multiple cortical tubers and/or radial migration lines	“Confetti” skin lesions
Subependymal nodules (≥ 2)	Sclerotic bone lesions
Subependymal giant cell astrocytomas	
Renal angiomyolipomas (≥ 2)**	
Cardiac rhabdomyoma	
Lymphangioleiomyomatosis**	

* *Identification of a clearly pathogenic genetic mutation that prevents protein synthesis and/or inactivates the function of the TSC1 or TSC2 proteins; other variants should be evaluated with caution.*

** *The combination of angiomyolipomas and lymphagioleiomyomatosis without other findings does not meet the diagnostic criteria.*

## Kidney Manifestations Related to TSC

**Figure 3:**
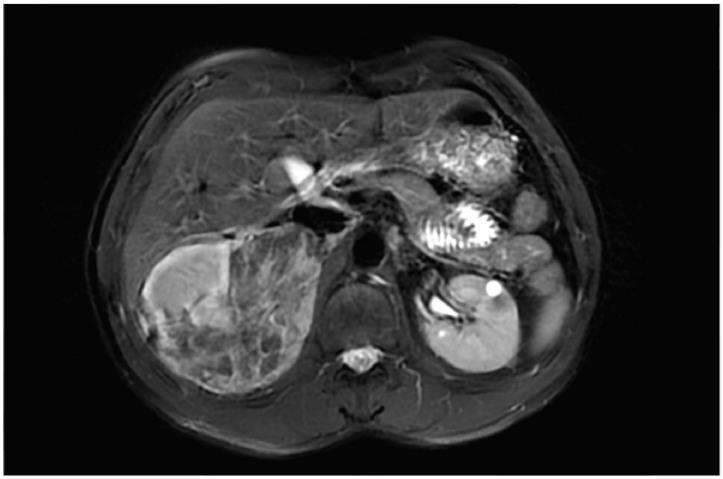
MRI of a male patient with tuberous sclerosis complexshowing a large angiomyolipoma in the right kidney and a cyst in the left kidney.

Renal involvement in TSC can begin in early childhood and remain asymptomatic or oligosymptomatic for several years^18^. Its incidence and severity increase over the life course, constituting a major cause of morbidity and mortality^6,18^. Approximately 80% of children will exhibit some renal manifestation by the age of 10, with lesions progressing throughout life^6,18^. The most prevalent renal manifestation is angiomyolipoma followed by renal cysts (Figure 3). Other renal tumors, such as renal cell carcinoma (RCC) and oncocytoma, can occur less frequently^18,19^.

### Renal Angiomyolipoma

Angiomyolipoma is present in over half TSC patients and up to 85% of those with kidney lesions. Onset can be early, affecting up to 20% of children under the age of 2^6,18,19^. The condition is characterized by varied contributions of adipocytes, smooth muscle cells, and endothelial cells, and genetic analysis has revealed that the second genetic hit is present in all three cell lineages in angiomyolipoma which indicates that the three elements arise from a common precursor cell that has undergone inactivation of both alleles, either *TSC1* or *TSC2* ^4,6,10^. The cell of origin of angiomyolipoma is believed to derive from renal pericytes, which are perivascular epithelial cells with high a capacity for differentiation, angiogenesis, and lipid accumulation^19^. Due to significant growth potential and abnormal vascularization, angiomyolipomas can develop intratumoral aneurysms, which are prone to spontaneous rupture and hemorrhages, particularly in lesions larger than 3 cm in diameter^6,16^. Renal complications have been considered to be one of the main causes of death in these patients^6^.

**Figure 4:**
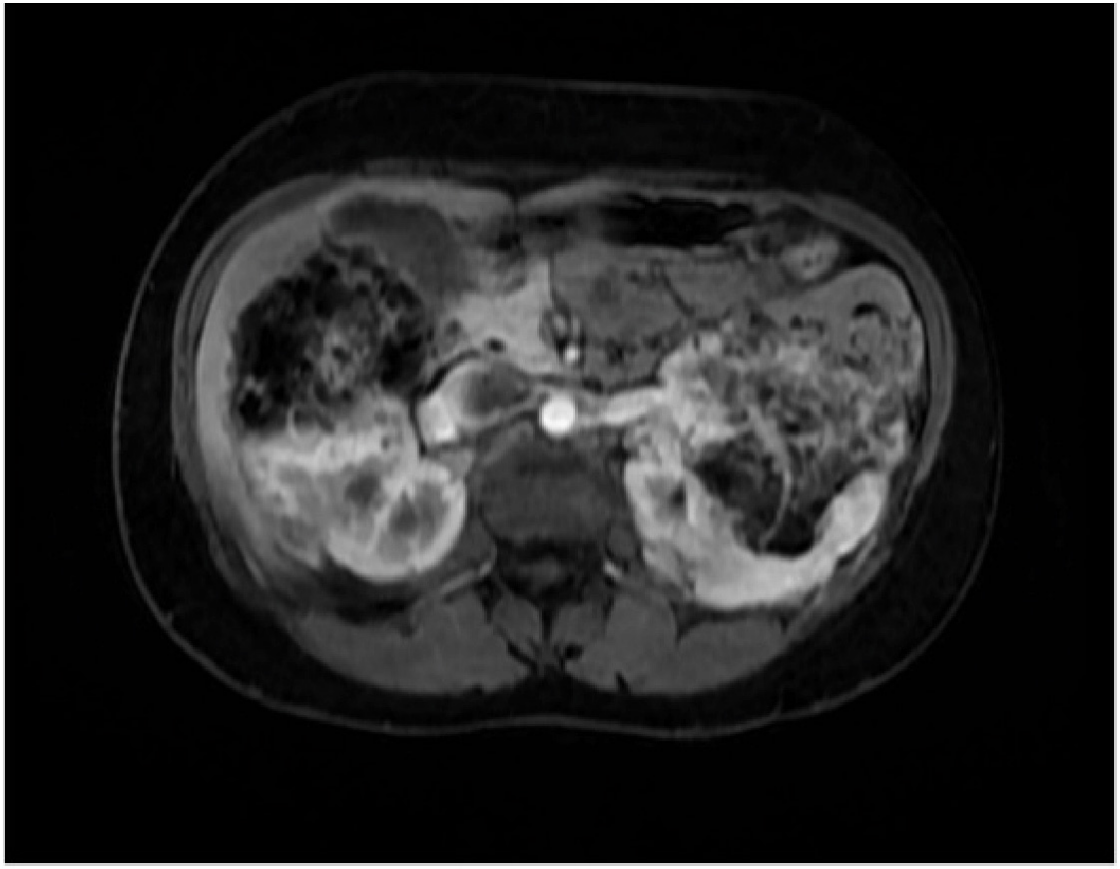
MRI showing large renal masses compatible with angiomyolipomas that replace practically the entire renal parenchyma in a patient with tuberous sclerosis complex.

The progressive destruction of renal parenchyma caused by the growth of the angiomyolipoma appears to play a pivotal role in the loss of renal function (Figure 4)^6,16^. Other factors such as compensatory glomerular hyperfiltration, arterial hypertension, repeated embolizations, nephrectomy, and the use of nephrotoxic medications can also contribute to this loss^4,20^. Approximately 40% of TSC patients experience a premature reduction in glomerular filtration rate (GFR). By the age of 50, they may have stage 3 or lower chronic kidney disease (CKD), compared to 3% of the general population^6^. Although the risk of progressing to dialytic stage 5 CKD (CKD-5D) is small (1–4% of patients progress to renal replacement therapy), an accelerated CKD-related cardiovascular disease poses a significant threat to these patients^4,6,17^.

### Cystic Kidney Disease

Renal cysts are the second most frequent renal manifestation associated with TSC^6,17^. They can emerge as early as childhood and are considered the second most common kidney lesion, affecting up to half of all patients in their lifetime^18^. Renal cysts can be single or multiple and are generally asymptomatic. However, they are susceptible to complications as observed in other renal cystic diseases, such as hemorrhage, infection, and pain^19,20^. In up to 2–3% of patients, large deletions in *TSC2* can affect the adjacent *PKD1* gene, both located on chromosome 16, resulting in the *PKD1/TSC2* contiguous deletion syndrome, characterized by severe, early-onset, and accelerated cystic disease. Individuals with this syndrome have multiple renal cysts identifiable at birth, changes in urinary concentration, hypertension, and rapid loss of renal function that can progress to CKD-5D in adolescence^6,19^.

The mechanism of formation of TSC-related renal cysts isn’t yet fully understood. Unlike angiomyolipoma, there is no loss of heterozygosity due to a second hit, and the expression of tuberin and hamartin has been observed in the cysts^21,22^. Experimental studies using a mouse model with knockdown *TSC2* gene suggest that extracellular vesicles play an important role in TSC-related cystogenesis^23,24,25^.

### Renal Cell Carcinoma and Oncocytoma

The incidence of renal cell carcinoma (RCC) in TSC is 2 to 4 times higher (2–4% vs 1%) and appears earlier in life (~30 vs ~55 years) than the general population^19^. RCCs are often multiple and bilateral, growing faster than angiomyolipoma, and typically lacking lipid content^26,27^. There is a predominance among women, contrasting once again with the general population where RCC is more common among men^26,27^. Various histological types of RCC have been described, including fibromyomatous stroma, solid and cystic eosinophilic, vacuolar eosinophilic, and low-grade oncocytic RCC^28^.

Lastly, oncocytoma is a rare benign tumor, usually unilateral and solitary, with a rapid growth and large, but without invasive behavior. The histological diagnosis of oncocytoma is based on the identification of intensely eosinophilic granular cells. Surgical resection remains the treatment of choice^19^.

## mTOR Inhibitors: A Specific Therapy for TSC

Advances in the understanding of the pathophysiology of TSC have paved the way for the utilization of mTOR inhibitors, *i.e.* sirolimus and everolimus, for the treatment of this disease. From a renal perspective, mTOR inhibitors promote growth control and hinder the progression of angiomyolipoma, reducing the need for surgical and invasive interventions such as nephrectomy and repeated embolizations, which lead to loss of renal mass and function^4,17,19^.

Experimental studies in a TSC animal model demonstrated that sirolimus treatment effectively inactivated the mTOR pathway resulting in a substantial reduction in renal tumors, improved survival, and enhanced clinical status^29^. Sirolimus’ impact on tumor response has been linked to apoptosis induction, decrease in cell size, and necrosis, possibly due to its pro-thrombotic or anti-angiogenic tumor effects^23,25,30^.

The initial clinical evidence supporting the potential benefits of sirolimus in TSC patients was reported in a 19-year-old patient in 2006^31^. Subsequently, an open label, non-randomized clinical study evaluated the effect of sirolimus on the reduction of angiomyolipoma volume in patients with TSC or sporadic LAM^32^. Of the 25 included patients, 20 completed the 24-month follow-up, encompassing an initial 12 months of treatment (initial dose 0.25 mg/m^2^ with adjustments to maintain serum levels at 10–15 ng/mL) followed by 12 months without medication. A mean reduction of approximately 50% in the baseline size of the angiomyolipoma and a tendency for volume increase after stopping the medication were observed by MRI^32^. Subsequent studies evaluating sirolimus have consistently demonstrated its efficiency in controlling renal angiomyolipoma. In an open multicenter non-randomized phase 2 study evaluating 16 patients with TSC or sporadic LAM and renal angiomyolipoma taking sirolimus for up to 24 months (serum level 3–10 ng/mL), a sustained reduction in the diameter of the angiomyolipoma was observed in all patients, with the greatest reduction in the first year of therapy^33^. Other phase 2 clinical studies have reported similar effects, accompanied by a decrease in serum levels of vascular endothelial growth factor D (VEGF-D)^34,35,36,37,38^. More recently, in a retrospective study, Watanabe et al. analyzed the computed tomography scans of 14 patients with TSC taking sirolimus, and demonstrated that the decrease in the size of the angiomyolipoma occurs mainly due to a reduction in the hypervascularized and fat-poor compartments^38^. They have also reported a pronounced reduction in the diameter of aneurysms and in intratumoral vascular ectasias^38^. Importantly, it should be mentioned that patients using sirolimus require close monitoring as they may experience adverse events such as stomatitis, skin lesions, dyslipidemia, and respiratory infection among others, whose frequency and severity commonly ameliorate overtime^32,33,39-42^.

Everolimus, an analog of sirolimus with greater oral availability and lower protein binding, was investigated for the treatment of TSC in the EXIST-1 study, which analyzed its effects in reducing SEGA^40^. Subsequently, a multicenter, randomized, double-blind, placebo-controlled phase 3 study (EXIST-2) evaluated the response of renal angiomyolipoma to everolimus (10 mg daily) in adult patients (N = 118) with TSC or sporadic LAM with angiomyolipoma ≥ 3 cm in diameter^41^. The median exposure was 38 weeks for the everolimus group and 34 weeks for the placebo group. The response rate in AML reduction, *i.e.* reduction of at least 50% of the total angiomyolipoma volume, was 42% for the everolimus group [33 of 79 (95% CI 31-53%)] and 0% for placebo, with a median response time to everolimus of 2 to 9 months^41^. The EXIST-2 extension study has demonstrated that the treatment effect is maintained over time, with an increase in the response rate compared to the primary study, from 42% to 54%. Moreover, approximately 97% of the patients showed a reduction in angiomyolipoma compared to baseline^42^. None of the patients using everolimus presented renal bleeding^42^. In addition, the *post hoc* analysis of pediatric patients (N = 33) from the EXIST-1 study demonstrated the safety and efficacy of everolimus in the treatment of renal angiomyolipoma in this population^43^. Importantly, the mean eGFR of patients from EXIST-1 (N = 111) and EXIST-2 (N = 112) studies using everolimus remained stable throughout the follow-up period. A decline in eGFR was observed only in some patients who already had significant alterations in pre-treatment renal function. In addition, the presence of proteinuria, assessed by urinary dipstick after starting everolimus, was considered mild in most cases^44^. Real-world data have confirmed the efficacy and safety of using everolimus at an average daily dose of 8.4 mg for the treatment of TSC-related renal angiomyolipoma, promoting regression and stabilization of the lesions for a period of up to 3 years^45^. Recently, the update of a Cochrane systematic review, which included patient data (N = 703) from six randomized clinical trials confirmed that everolimus may reduce angiomyolipoma size by 50% (relative risk 24.69, 95% CI 3.51 to 173.41; P = 0.001), in addition to reducing the size of SEGA and the frequency of seizures and improving skin lesions^46^. Although no difference was observed in the total number of adverse effects between the treatment and placebo groups, more participants in the former required dose reduction, interruption, or discontinuation of medication, and had more serious adverse effects^46^.

Studies have consistently reported that mTOR inhibitors are associated with controlling the growth of angiomyolipoma, reducing both its size and the risk of bleeding and the need for surgical intervention, while preserving renal function with a relatively low incidence of serious adverse effects^32–47,48^. Given that mTOR inhibitors, since they have only cytostatic effects, must be used indefinitely, perhaps for life, to guarantee their beneficial effects, it is important to be aware of their adverse effects (Chart 2) and to devise strategies to identify and manage them appropriately. Regular laboratory and clinical monitoring of potential adverse effects, patient education and, whenever necessary, dose reduction or temporary suspension of medication, are essential part of patient care^49^. In addition, aiming to reduce the occurrence of side effects, new protocols for the use of mTOR inhibitors for the treatment of TSC have been evaluated. A recent prospective study has compared the use of everolimus at a standard dose (N = 23; 10 mg/day for 12 months) with sequential dosing (N = 30; 10 mg/day for 4 months, followed by 5 mg/day until the 12th month)^50^. The sequential treatment group showed similar efficacy to the standard dose, with a lower incidence of adverse effects and lower cost^50^. Furthermore, in a prospective study with a 48-month follow-up (N = 11) in which everolimus was started at lower doses of 2.5 mg/day and increased to 5 mg/day according to tolerance and serum level (8–15 ng/mL), angiomyolipoma tumor mass decreased from the sixth month of treatment and remained stable throughout the study^51^.

**Chart 2:** Main adverse effects related to the use of mTOR inhibitors sirolimus and everolimus

	Everolimus	Sirolimus
**Infectious**	Upper respiratory tract, urinary tract infection, pneumonia	Upper respiratory tract, pneumonia, cellulitis, urinary infection
**Hematological**	Leukopenia, anemia	Leukopenia, anemia
**Metabolic**	Dyslipidemia, hypophosphatemia	Dyslipidemia, hypokalemia
**Neurological**	Headache, seizure	Headache, dizziness, tremor
**Gastrointestinal**	Stomatitis, abdominal pain, nausea, vomiting	Stomatitis, diarrhea, nausea, and abdominal pain
**Dermatological**	Acne, eczema	Acne, folliculitis
**Gynecological**	Amenorrhea, menstrual irregularity	Amenorrhea, menstrual irregularity
**General**	Arthralgia, fatigue	Peripheral edema, fatigue
**Laboratorial**	Increase in LDH	Increased LDH, AST, and ALT
**Renal**	Proteinuria	Proteinuria
**Cardiac**		Tachycardia, high blood pressure

* *LDH: lactate dehydrogenase enzyme; AST: glutamic-oxalacetic transaminase; ALT: glutamic-pyruvic transaminase.*

**Figure 5:**
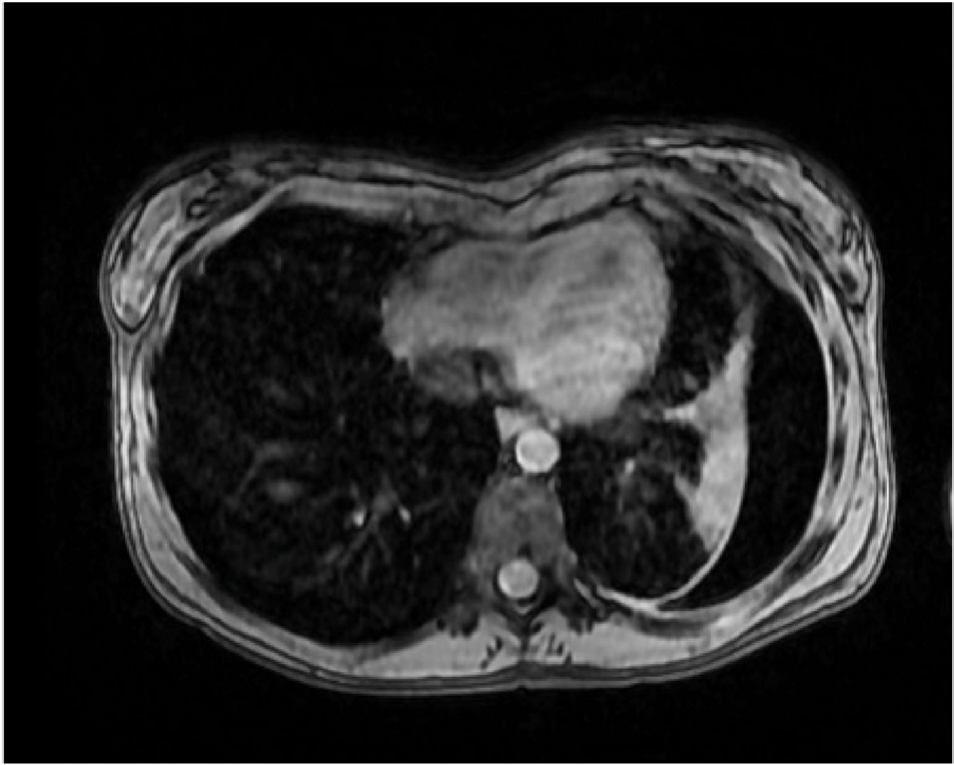
MRI of a female patient with tuberous sclerosis complex and lymphangioleiomyomatosis. Pneumothorax, which is a complication of lymphangioleiomyomatosis, with ipsilateral pulmonary atelectasis is noted.

Another important issue on the use of mTOR inhibitors in TSC that deserves our attention is whether one of the commercially available drugs, sirolimus or everolimus, is superior to the other. As they have a similar molecular structure, it is reasonable to assume that both may provide alike benefits for TSC patients^52^. Unfortunately, the lack of randomized clinical trials evaluating sirolimus for the treatment of TSC-related renal angiomyolipoma has become a barrier to the approval of this medication by regulatory agencies for this purpose. Recently, a multicenter retrospective study has suggested that both medications are good therapeutic options, with everolimus appearing to be somewhat superior in reducing TSC-related renal angiomyolipoma^53^. Everolimus was approved by the Food and Drug Administration (FDA) and the European Medicines Agency (EMA) for the treatment of renal angiomyolipoma ≥ 3 cm in diameter in adult patients with TSC in 2016 and 2018, respectively^54,55^. It is currently considered the first-line therapy for the elective treatment of angiomyolipoma^17^. In Brazil, sirolimus was incorporated into the Brazilian national health system for the treatment of LAM (SCTIE/MS Ordinance No. 24 of August 4, 2020) (Figure 5)^56^. The National Health Surveillance Agency (ANVISA) currently authorizes the marketing and use of everolimus for the treatment of angiomyolipoma associated with TSC^57^.

In contrast to the convincing data on the efficacy of mTOR inhibitors in patients with angiomyolipoma, the efficacy of these agents in the treatment of renal cystic disease associated with TSC has yet to be fully defined, but they have shown promising results in recent research^20,58^. Moreover, even though everolimus has been used as a treatment option for advanced CRC in the general population^59,60^, the efficacy of mTOR inhibitors for RCC in TSC remains uncertain^61,62,63,64^.

## Nephrological Care and Follow-Up in the TSC

Patients with TSC require nephrological follow-up as an integral part of the multidisciplinary care from childhood through adulthood to ensure early diagnosis of renal alterations, periodic monitoring of renal function, and the initiation of a specific treatment when indicated. According to the latest international consensus on TSC, it is recommended to perform imaging tests, preferably MRI, to assess renal lesions such as angiomyolipoma and cysts in newly diagnosed or suspected patients. Ideally, MRI should be repeated every 1 to 3 years to evaluate lesion growth and progression, irrespective of the therapeutic modality^17^. Distinguishing RCCs from angiomyolipoma, especially those with low fat content, can be challenging^19^. Although various techniques help in this differentiation, rapid lesion growth of pre-existing lesions and changes in cyst morphology suggest potential malignancy. MRI stands out as the gold standard imaging method for diagnosis^20^. In cases where MRI is inconclusive, a biopsy may be necessary^17,19^.

Glomerular filtration rate, estimated from serum creatinine and/or cystatin C, and proteinuria should be monitored at least once a year^17^. Hypertension is more common in TSC patients than in the general population. Hence, blood pressure control is essential and may help to slow down the progression of CKD^6^. Angiotensin-converting enzyme inhibitors and angiotensin receptor blockers are the preferred drugs for this purpose, as they might have a suppressive effect on tumor growth on AML and cysts^6^.

For specific therapy, mTOR inhibitors are the first-line treatment for asymptomatic patients with angiomyolipomas > 3 cm in diameter^17^. Lower doses of everolimus than those recommended by the EXIST-2 study, such as 5 mg/day, have shown good therapeutic responses^17,51^. Selective embolization and partial nephrectomy are potential second-line therapies for asymptomatic angiomyolipoma larger than 3 cm in diameter^17^. In cases of AML with acute hemorrhage, arterial embolization, followed by corticotherapy to prevent post-embolization syndrome are indicated^17^.

In terms of renal replacement therapy, all therapeutic modalities can be considered^17^. Kidney transplantation is a viable option, boasting higher graft survival rates than in the general population^65^. It is noteworthy that mTOR inhibitors can be beneficial in preventing tumor bleeding even in advanced CKD. Therefore, their use should be contemplated in TSC patients on dialysis therapy and included in the immunosuppressive regimen for kidney transplantation^66,67,68^. Finally, the treatment of RCC is tailored to tumor staging and encompasses various therapeutic modalities, such as radiotherapy, immunotherapy, and/or surgical resection^69^. Chart 3 summarizes the main recommendations for managing TSC-related AML.

**Chart 3:** Main recommendations for the nephrological care of patients with TSC

Diagnosis and follow-up		
Blood pressure control	Carry out follow-up according to current guidelines since the TSC diagnosis. Give preference to the use of ACEI/ARB.
eGFR	At least once a year
Proteinuria	At least once a year
Imaging exam (preferably MRI)	Perform when TSC is diagnosed or suspected to aid in diagnostic confirmation. Repeat every 1 to 3 years to assess growth and progression of the lesions.
**Angiomyolipomas management**	
Asymptomatic patients with angiomyolipomas < 3 cm in diameter	Follow-up
Asymptomatic patients, > 18 years old, with angiomyolipomas > 3 cm in diameter	First-line therapy: mTOR inhibitors. Monitor dose according to adverse effects and serum level. Therapeutic options: partial nephrectomy and selective arterial embolization
Angiomyolipomas with acute bleeding	First-line therapy: arterial embolization followed by systemic corticotherapy for 7 days (to avoid post-embolization syndrome). If possible, avoid nephrectomy.
**Renal replacement therapy at the TSC**	
RRT modality	All modalities can be used. Consider maintaining the use of mTOR inhibitors for dialysis patients to prevent angiomyolipomas bleeding in native kidneys and, if necessary, control other manifestations of the disease.
Kidney transplant	Consider the use of mTOR inhibitors in the immunosuppressive regimen, also to prevent angiomyolipomas bleeding in native kidneys and, if necessary, to control other manifestations of the disease. Consider nephrectomy of native kidneys depending on kidney size and angiomyolipomas due to risk of bleeding.

* *TSC: tuberous sclerosis complex; ACEI: angiotensin-converting enzyme inhibitors; ARB: angiotensin receptor blockers; eGFR: estimated glomerular filtration rate; RRT: renal replacement therapy.*

## Future Perspectives

The identification of the mTOR pathway’s involvement in the pathophysiology of TSC-related angiomyolipoma has enhanced our understanding of the disease. Nevertheless, therapeutic options remain limited, primarily exerting a cytostatic effect. Further research is warranted to identify biomarkers, novel therapeutic targets, and cytotoxic alternatives for effective disease control. In this regard, a recent randomized double-blind placebo-controlled clinical trial investigated metformin as a potential therapeutic option for TSC-related tumors, due to its inhibitory mechanism of the mTOR pathway through the activation of adenosine monophosphate-activated protein kinase (AMPK) and p53. While it exhibited promising results in reducing the volume of SEGA, a similar effect was not observed with renal angiomyolipoma^70^.

*In vitro* studies and animal models suggest prospective therapeutic targets. The mTOR pathway’s influence on vitamin A metabolism and the expression of retinoic acid receptor beta (RARβ) has been noted^71^. An *in vitro* study using cell lines with pathogenic variants in *TSC* showed lower expression of RARβ. Retinoic acid, a vitamin A metabolite already used to treat some types of leukemia, was able to normalize RARβ levels and limit cell migration. Although there was no significant effect on cell proliferation, the study suggests that combining mTOR inhibitors with retinoic acid could be a therapeutic option to reduce the doses and side effects of these medications^71^. Studies evaluating the potential effect of tyrosine kinase inhibitors imatinib and nilotinib in TSC have shown a cytotoxic effect of these drugs on both angiomyolipoma and LAM cell lines, as well as a reduction in tumor growth in animal models treated with imatinib^72^.

Gene therapy seems to be a promising field. A preclinical study with animal models affected by *TSC1* mutations and central nervous system lesions showed increased survival and restoration of protein functions after the intravenous administration of an adenovirus viral vector encoding for hamartin^73^. Furthermore, it has also been demonstrated that the use of an adenovirus viral vector coded for tuberin in animal model affected by *TSC2* improved survival and reduced brain involvement^74^.

Last but not least, mitochondrial regulation and function of affected cell lines^75^, activation of p53 and regulation of apoptosis^76^, inflammatory mediators of the cellular microenvironment^77^, involvement of circular RNA in tumorigenesis^78^, and altered interaction between *TSC2* gene proteins and high-density lipoprotein-binding proteins^79^ have been considered potential therapeutic targets in TSC-related angiomyolipoma.

## Conclusions

The high prevalence of kidney involvement in TSC, coupled with its association with high morbidity and mortality, underscores the importance of providing nephrological care for these patients from childhood onward. The understanding of the pathophysiology of TSC was crucial to the use of mTOR inhibitors to treat this disorder, opening avenues for the identification of novel therapeutic targets and medicines with higher efficacy and fewer adverse effects to modify the course of the disease. Ongoing and future research aiming to prevent the onset and progression of TSC-related manifestations, including renal lesions, has given hope for a better life to the persons affected by TSC and their relatives.

## References

[1] Rodriguez Gómez M (1995). History of the tuberous sclerosis complex. Brain Dev.

[2] Bourneville DM (1880). Sclerose tubéreuse des circonvolutions cerebrales: idiotie et épilesie hémiplégique. Arch Neurol (Paris).

[3] Northrup H, Krueger DA, Roberds S, Smith K, Sampson J, Korf B (2013). Tuberous sclerosis complex diagnostic criteria update: recommendations of the 2012 international tuberous sclerosis complex consensus conference. Pediatr Neurol.

[4] Lam HC, Siroky BJ, Henske EP (2018). Renal disease in tuberous sclerosis complex: pathogenesis and therapy. Nat Rev Nephrol.

[5] Curatolo P, Bombardieri R, Jozwiak S (2008). Tuberous sclerosis. Vol. 372: The Lancet.

[6] Bissler JJ, Kingswood J (2018). Renal manifestation of tuberous sclerosis complex. Am J Med Genet C Semin Med Genet.

[7] Rakowski SK, Winterkorn EB, Paul E, Steele DJR, Halpern EF, Thiele EA (2006). Renal manifestations of tuberous sclerosis complex: incidence, prognosis, and predictive factors. Kidney Int.

[8] Faucz Munhoz da Cunha M, Sevignani G, Memari Pavanelli G, de Carvalho M, Carvalho Barreto F (2020). Rare inherited kidney diseases: an evolving field in nephrology. J Bras Nefrol.

[9] Monich AG, Cunha MFM, Barreto FC (2023). mTOR inhibitors are the first-choice therapy for renal angiomyolipomas secondary to tuberous sclerosis. J Nephrol (J. Bras. Nefrol.).

[10] Caban C, Khan N, Hasbani DM, Crino PB (2017). Genetics of tuberous sclerosis complex: implications for clinical practice. Vol. 10: Application of Clinical Genetics.

[11] Azzi-Nogueira D (2016). Os produtos dos genes TSC1 e TSC2 em processos neurodegenerativos [tese].

[12] Knudson AG (1971). Mutation and cancer: statistical study of retinoblastoma. Proc Natl Acad Sci USA.

[13] Henske EP, Neumann HPH, Scheithauer BW, Herbst EW, Short MP, Kwiatkowski DJ (1995). Loss of Heterozygosity in the Tuberous Sclerosis (TSCZ) Region of Chromosome Band l6p13 Occurs in Sporadic as Well as TSC-Associated Renal Angiomyolipomas. Genes Chromosomes Cancer.

[14] Tyburczy ME, Jozwiak S, Malinowska IA, Chekaluk Y, Pugh TJ, Wu CL (2015). A shower of second hit events as the cause of multifocal renal cell carcinoma in tuberous sclerosis complex. Hum Mol Genet.

[15] Leiden Open Variation Database [Internet] (2023). https://www.lovd.nl/.

[16] Kingswood JC, Bruzzi P, Curatolo P, De Vries PJ, Fladrowski C, Hertzberg C (2014). TOSCA - first international registry to address knowledge gaps in the natural history and management of tuberous sclerosis complex. Orphanet J Rare Dis.

[17] Northrup H, Aronow ME, Bebin EM, Bissler J, Darling TN, de Vries PJ (2021). Updated international tuberous sclerosis complex diagnostic criteria and surveillance and management recommendations. Pediatr Neurol.

[18] Kingswood JC, Belousova E, Benedik MP, Carter T, Cottin V, Curatolo P (2019). Renal angiomyolipoma in patients with tuberous sclerosis complex: findings from the TuberOus SClerosis registry to increase disease Awareness. Nephrol Dial Transplant.

[19] Trnka P, Kennedy SE (2021). Renal tumors in tuberous sclerosis complex. Pediatr Nephrol.

[20] Gallo-Bernal S, Kilcoyne A, Gee MS, Paul E (2023). Cystic kidney disease in tuberous sclerosis complex: current knowledge and unresolved questions. Pediatr Nephrol.

[21] Wilson C, Bonnet C, Guy C, Idziaszczyk S, Colley J, Humphreys V (2006). Tsc1 haploinsufficiency without mammalian target of rapamycin activation is sufficient for renal cyst formation in Tsc1 +/− Mice. Cancer Res.

[22] Bonsib SM, Boils C, Gokden N, Grignon D, Gu X, Higgins JPT (2016). Tuberous sclerosis complex: hamartin and tuberin expression in renal cysts and its discordant expression in renal neoplasms. Pathol Res Pract.

[23] Kumar P, Zadjali F, Yao Y, Johnson D, Siroky B, Astrinidis A (2022). Tsc2 mutation induces renal tubular cell nonautonomous disease. Genes Dis.

[24] Kumar P, Zadjali F, Yao Y, Bissler JJ (2021). Renal cystic disease in tuberous sclerosis complex. Experimental Biology and Medicine.

[25] Zadjali F, Kumar P, Yao Y, Johnson D, Astrinidis A, Vogel P (2020). Tuberous sclerosis complex axis controls renal extracellular vesicle production and protein content. Int J Mol Sci.

[26] Guo J, Tretiakova MS, Troxell ML, Osunkoya AO, Fadare O, Sangoi AR (2014). Tuberous Sclerosis–associated Renal Cell Carcinoma: a clinicopathologic study of 57 separate carcinomas in 18 patients. Am J Surg Pathol.

[27] Yang P, Cornejo KM, Sadow PM, Cheng L, Wang M, Xiao Y (2014). Renal cell carcinoma in tuberous sclerosis complex. Am J Surg Pathol.

[28] Kapur P, Brugarolas J, Trpkov K (2023). Recent advances in renal tumors with tsc/mtor pathway abnormalities in patients with tuberous sclerosis complex and in the sporadic setting. Cancers (Basel).

[29] Kenerson H, Dundon TA, Yeung RS (2005). Effects of rapamycin in the eker rat model of tuberous sclerosis complex. Pediatr Res.

[30] Guba M, Yezhelyev M, Eichhorn ME, Schmid G, Ischenko I, Papyan A (2005). Rapamycin induces tumor-specific thrombosis via tissue factor in the presence of VEGF. Blood.

[31] Wienecke R, Fackler I, Linsenmaier U, Mayer K, Licht T, Kretzler M (2006). Antitumoral activity of rapamycin in renal angiomyolipoma associated with tuberous sclerosis complex. Am J Kidney Dis.

[32] Bissler JJ, McCormack FX, Young LR, Elwing JM, Chuck G, Leonard JM (2008). Sirolimus for angiomyolipoma in tuberous sclerosis complex or lymphangioleiomyomatosis. N Engl J Med.

[33] Davies DM, De Vries PJ, Johnson SR, McCartney DL, Cox JA, Serra AL (2011). Sirolimus therapy for angiomyolipoma in tuberous sclerosis and sporadic lymphangioleiomyomatosis: a phase 2 trial. Clin Cancer Res.

[34] Dabora SL, Franz DN, Ashwal S, Sagalowsky A, DiMario FJ, Miles D (2011). Multicenter phase 2 trial of sirolimus for tuberous sclerosis: kidney angiomyolipomas and other tumors regress and VEGF- D levels decrease. PLoS One.

[35] Cabrera-López C, Martí T, Catalá V, Torres F, Mateu S, Ballarín J (2012). Assessing the effectiveness of rapamycin on angiomyolipoma in tuberous sclerosis: a two years trial. Orphanet J Rare Dis.

[36] Cabrera-López C, Martí T, Catalá V, Torres F, Mateu S, Castán JB (2011). Efectos de la rapamicina en los angiomiolipomas de pacientes con esclerosis tuberosa. Nefrologia.

[37] Malinowska IA, Lee N, Kumar V, Thiele EA, Franz DN, Ashwal S (2013). Similar trends in serum VEGF-D levels and kidney angiomyolipoma responses with longer duration sirolimus treatment in adults with tuberous sclerosis. PLoS One.

[38] Watanabe EH, Coelho FMA, Leão Fo H, Balbo BEP, Neves PDMM, Franzin FM (2021). The effect of sirolimus on angiomyolipoma is determined by decrease of fat-poor compartments and includes striking reduction of vascular structures. Sci Rep.

[39] Peng ZF, Yang L, Wang TT, Han P, Liu ZH, Wei Q (2014). Efficacy and safety of sirolimus for renal angiomyolipoma in patients with tuberous sclerosis complex or sporadic lymphangioleiomyomatosis: a systematic review. J Urol.

[40] Krueger DA, Care MM, Holland K, Agricola K, Tudor C, Mangeshkar P (2010). Everolimus for subependymal giant-cell astrocytomas in tuberous sclerosis. N Engl J Med.

[41] Bissler JJ, Kingswood JC, Radzikowska E, Zonnenberg BA, Frost M, Belousova E (2013). Everolimus for angiomyolipoma associated with tuberous sclerosis complex or sporadic lymphangioleiomyomatosis (EXIST-2): A multicentre, randomised, double-blind, placebo-controlled trial. Lancet.

[42] Bissler JJ, Kingswood JC, Radzikowska E, Zonnenberg BA, Frost M, Belousova E (2016). Everolimus for renal angiomyolipoma in patients with tuberous sclerosis complex or sporadic lymphangioleiomyomatosis: extension of a randomized controlled trial. Nephrol Dial Transplant.

[43] Bissler JJ, Franz DN, Frost MD, Belousova E, Bebin EM, Sparagana S (2018). The effect of everolimus on renal angiomyolipoma in pediatric patients with tuberous sclerosis being treated for subependymal giant cell astrocytoma. Pediatr Nephrol.

[44] Bissler JJ, Budde K, Sauter M, Franz DN, Zonnenberg BA, Frost MD (2019). Effect of everolimus on renal function in patients with tuberous sclerosis complex: evidence from EXIST-1 and EXIST-2. Nephrol Dial Transplant.

[45] Chung NKX, Metherall P, McCormick JA, Simms RJ, Ong ACM (2022). Individualized everolimus treatment for tuberous sclerosis-related angiomyolipoma promotes treatment adherence and response. Clin Kidney J.

[46] Sasongko TH, Ismail NFD, Zabidi-Hussin Z (2016). Rapamycin and rapalogs for tuberous sclerosis complex. Cochrane Database Syst Rev.

[47] Bissler JJ, Kingswood JC, Radzikowska E, Zonnenberg BA, Belousova E, Frost MD (2017). Everolimus long-term use in patients with tuberous sclerosis complex: four-year update of the EXIST-2 study. PLoS One.

[48] Li M, Zhou Y, Chen C, Yang T, Zhou S, Chen S (2019). Efficacy and safety of mTOR inhibitors (rapamycin and its analogues) for tuberous sclerosis complex: A meta-analysis. Orphanet J Rare Dis.

[49] Davies M, Saxena A, Kingswood JC (2017). Management of everolimus-associated adverse events in patients with tuberous sclerosis complex: a practical guide. Orphanet J Rare Dis.

[50] Gu L, Peng C, Zhang F, Fang C, Guo G (2021). Sequential everolimus for angiomyolipoma associated with tuberous sclerosis complex: a prospective cohort study. Orphanet J Rare Dis.

[51] Wei CC, Tsai JD, Sheu JN, Chen SL, Tsao TF, Yang SH (2019). Continuous low-dose everolimus shrinkage tuberous sclerosis complex-associated renal angiomyolipoma: A 48-month follow-up study. J Investig Med.

[52] MacKeigan JP, Krueger DA (2015). Differentiating the mTOR inhibitors everolimus and sirolimus in the treatment of tuberous sclerosis complex. Neuro-oncol.

[53] Luo C, Zhang YS, Zhang MX, Chen MF, Li Y, Qi L (2021). Everolimus versus sirolimus for angiomyolipoma associated with tuberous sclerosis complex: a multi-institutional retrospective study in China. Orphanet J Rare Dis.

[54] Food and Drug Administration (2016). Afinitor [Internet].

[55] European Medicines Agency (2018). Votubia (everolimus) [Internet].

[56] Brasil. Agência Nacional de Vigilância Sanitária (2020). Portaria SCTIE/MS no 24 de 04 de agosto de 2020. Torna pública a decisão de ampliar o uso do sirolimo para o tratamento de indivíduos adultos com linfangioleiomiomatose (LAM), no âmbito do Sistema Único de Saúde – SUS, conforme Protocolo do Ministério da Saúde.

[57] Brasil. Agência Nacional de Vigilância Sanitária (2021). Everolimo [Internet].

[58] Siroky BJ, Towbin AJ, Trout AT, Schäfer H, Thamann AR, Agricola KD (2017). Improvement in renal cystic disease of tuberous sclerosis complex after treatment with mammalian target of rapamycin inhibitor. J Pediatr.

[59] Motzer RJ, Barrios CH, Kim TM, Falcon S, Cosgriff T, Harker WG (2014). Phase II randomized trial comparing sequential first-line everolimus and second-line sunitinib versus first-line sunitinib and second-line everolimus in patients with metastatic renal cell carcinoma. J Clin Oncol.

[60] Motzer RJ, Escudier B, McDermott DF, George S, Hammers HJ, Srinivas S (2015). Nivolumab versus everolimus in advanced renal-cell carcinoma. N Engl J Med.

[61] Faes S, Demartines N, Dormond O (2021). Mechanistic target of rapamycin inhibitors in renal cell carcinoma: potential, limitations, and perspectives. Front Cell Dev Biol.

[62] Alsidawi S, Kasi PM (2018). Exceptional response to everolimus in a novel tuberous sclerosis complex-2 mutation-associated metastatic renal-cell carcinoma. Cold Spring Harb Mol Case Stud.

[63] Kwiatkowski DJ, Manning BD (2014). Molecular basis of giant cells in tuberous sclerosis complex. N Engl J Med.

[64] El-Hashemite N, Zhang H, Henske EP, Kwiatkowski DJ (2003). Mutation in TSC2 and activation of mammalian target of rapamycin signalling pathway in renal angiomyolipoma. Lancet.

[65] Vabret E, Couchoud C, Lassalle M, Vigneau C (2021). From tuberous sclerosis complex to end stage renal disease: who are these patients?. J Nephrol.

[66] Somers MJG, Paul E (2015). Safety considerations of mammalian target of rapamycin inhibitors in tuberous sclerosis complex and renal transplantation. J Clin Pharmacol.

[67] Balligand JL, Pirson Y, Squifflet JP, Cosyns JP, Alexandre GPG, Strihou CY (1990). Outcome of patients with tuberous sclerosis after renal transplantation. Transplantation.

[68] Tarasewicz A, De¸bska-S´lizien´ A, Konopa J, Zdrojewski Z, Rutkowski B (2009). Rapamycin as a therapy of choice after renal transplantation in a patient with tuberous sclerosis complex. Transplant Proc.

[69] Ambalavanan M, Geller JI (2019). Treatment of advanced pediatric renal cell carcinoma. Pediatr Blood Cancer.

[70] Amin S, Mallick AA, Edwards H, Cortina-Borja M, Laugharne M, Likeman M (2021). The metformin in tuberous sclerosis (MiTS) study: a randomised double-blind placebo-controlled trial. EClinicalMedicine.

[71] Abdelwahab EMM, Bovari-Biri J, Smuk G, Harko T, Fillinger J, Moldvay J (2021). Normalization of enzyme expression and activity regulating Vitamin A metabolism increases RAR-Beta expression and reduces cellular migration and proliferation in diseases caused by tuberous sclerosis gene mutations. Front Oncol.

[72] Unachukwu U, Sonett J, Woode D, Shiomi T, Chada K, D’Armiento JM (2023). Tyrosine kinase inhibitors diminish renal neoplasms in a tuberous sclerosis model via induction of apoptosis. Mol Cancer Ther.

[73] Prabhakar S, Cheah PS, Zhang X, Zinter M, Gianatasio M, Hudry E (2019). Long-term therapeutic efficacy of intravenous AAV-Mediated hamartin replacement in mouse model of tuberous sclerosis Type 1. Mol Ther Methods Clin Dev.

[74] Cheah PS, Prabhakar S, Yellen D, Beauchamp RL, Zhang X, Kasamatsu S (2021). Gene therapy for tuberous sclerosis complex Type 2 in a mouse model by delivery of AAV9 encoding a condensed form of tuberin. Sci Adv.

[75] Abdelwahab EMM, Pal S, Kvell K, Sarosi V, Bai P, Rue R (2019). Mitochondrial dysfunction is a key determinant of the rare disease lymphangioleiomyomatosis and provides a novel therapeutic target. Oncogene.

[76] Abdelwahab EMM, Bovari-Biri J, Smuk G, Fillinger J, McPhail D, Krymskaya VP (2021). Activated p53 in the anti-apoptotic milieu of tuberous sclerosis gene mutation induced diseases leads to cell death if thioredoxin reductase is inhibited. Apoptosis.

[77] Zhan W, Xiaoyan L, Wenda W, Jing W, Seery S, Jiyu X (2022). A multi-omics study of diagnostic markers and the unique inflammatory tumor micro-environment involved in tuberous sclerosis complex-related renal angiomyolipoma. Int J Oncol.

[78] Zhao Y, Guo H, Wang W, Zheng G, Wang Z, Wang X (2021). High-throughput screening of circRNAs reveals novel mechanisms of tuberous sclerosis complex-related renal angiomyolipoma. Hum Genomics.

[79] Kosmas K, Filippakis H, Khabibullin D, Turkiewicz M, Lam HC, Yu J (2021). TSC2 interacts with HDLBP/vigilin and regulates stress granule formation. Mol Cancer Res.

