# Supplementary material for: Tuberous Sclerosis Complex and the kidneys: what nephrologists need to know
Source: J Bras Nefrol. 2024 Jul 5;46(3):e20240013. doi: 10.1590/2175-8239-JBN-2024-0013en (PMC11239183; doi:10.1590/2175-8239-JBN-2024-0013en)
Supplement: Translation

# Translation: Complexo da Esclerose Tuberosa e rins: o que os nefrologistas devem saber

**DOI:** 10.1590/2175-8239-JBN-2024-0013pt

**Authors:** Aline Grosskopf Monich, John J. Bissler, Fellype Carvalho Barreto

**Affiliations:** 1 Universidade Federal do Paraná, Departamento de Clínica Médica, Programa de Pós-Graduação em Medicina Interna e Ciências da Saúde, Curitiba, PR, Brasil.; 2 Hospital Universitário Evangélico Mackenzie, Serviço de Nefrologia, Curitiba, PR, Brasil.; 3 University of Tennessee, Health Science Center, Le Bonheur Children's Hospital, Department of Pediatrics, Memphis, TN, EUA.; 4 Le Bonheur Children's Hospital, Children's Foundation Research Institute, Memphis, TN, EUA.; 5 St. Jude Children’s Research Hospital, Pediatric Medicine Department, Memphis, TN, EUA.; 6 Universidade Federal do Paraná, Departamento de Clínica Médica, Serviço de Nefrologia, Curitiba, PR, Brasil.

**Keywords:** Esclerose Tuberosa, Angiomiolipoma, Inibidores de MTOR, Insuficiência Renal Crônica

## Abstract

O complexo da esclerose tuberosa (CET) é uma doença autossômica dominante caracterizada pelo desenvolvimento de hamartomas no sistema nervoso central, coração, pele, pulmões e rins e outras manifestações, incluindo convulsões, tubérculos corticais, linhas de migração radial, autismo e deficiência cognitiva. A doença está associada a variantes patogênicas nos genes *TSC1* ou *TSC2*, resultando na hiperativação da via mTOR, um importante regulador do crescimento e metabolismo celular. Consequentemente, a hiperativação da via mTOR leva à proliferação anormal do tecido e ao desenvolvimento de tumores sólidos. O envolvimento renal no CET é caracterizado pelo desenvolvimento de lesões císticas, carcinoma de células renais e angiomiolipomas renais, que podem progredir e causar dor, sangramento e perda da função renal. Nos últimos anos, houve uma mudança notável na abordagem terapêutica do CET, especialmente no tratamento das manifestações renais. Os inibidores de mTOR surgiram como a principal opção terapêutica, enquanto intervenções cirúrgicas como nefrectomia e embolização são reservadas principalmente para complicações que não respondem ao tratamento clínico, como hemorragia renal grave. Esta revisão se concentra nas principais características clínicas do CET, nos mecanismos subjacentes ao envolvimento renal, nos recentes avanços na terapia para lesões renais e nas perspectivas futuras.

## Introdução

Os primeiros relatos da doença reconhecida atualmente como complexo da esclerose tuberosa (CET) remontam a 1835, por meio de ilustrações de Pierre François Olive Rayer, mostrando lesões papulares no rosto de um homem^1^. Posteriormente, em 1862, Friedrich Daniel von Recklinghausen apresentou um caso à Sociedade Obstétrica de Berlim envolvendo um recém-nascido com múltiplos tumores cardíacos e esclerose cerebral^1^. No entanto, foi Désiré-Magloire Bourneville que, em 1880, forneceu a primeira descrição detalhada do envolvimento do sistema nervoso central, nomeando oficialmente a doença como esclerose tuberosa^1,2^.

O CET é uma doença autossômica dominante marcada pela formação de hamartomas em vários órgãos, incluindo rins, cérebro, pulmões, coração e pele. Além disso, outros achados e sintomas neurológicos, como tubérculos corticais, linhas de migração radial, convulsões, comprometimento cognitivo e autismo, podem estar presentes^3–6^. A incidência do CET varia de 1/6.000 a 1/10.000 nascidos vivos, com uma estimativa de 2.000.000 de indivíduos atualmente afetados pela doença em todo o mundo^4^. Embora a prevalência do CET seja consistente entre populações, etnias e sexos, certas manifestações, como a linfangioleiomiomatose pulmonar (LAM) e o angiomiolipoma renal (AML), parecem ser mais acentuadas em mulheres, sugerindo influência hormonal^7^. O envolvimento renal pode se desenvolver desde a infância. Com o passar do tempo, a progressão da lesão renal, principalmente o angiomiolipoma, acaba levando à hemorragia renal, intervenções cirúrgicas de emergência e diminuição da função renal^3,6^. A introdução dos inibidores de mTOR (mTOR) no final dos anos 2000 mudou a história natural do CET, melhorando significativamente a qualidade de vida e as taxas de sobrevida dos indivíduos afetados^4,6^.

Apesar dos recentes avanços no diagnóstico molecular e no desenvolvimento de terapias direcionadas, os pacientes com doenças raras, como o CET, ainda são negligenciados^8^. No Brasil, os dados epidemiológicos sobre o CET são escassos. Achados iniciais de um estudo observacional sobre o envolvimento renal no CET revelaram que apenas 25% dos pacientes estavam fazendo uso de inibidores de mTOR, e a nefrectomia parcial ou total ainda é realizada rotineiramente nesses pacientes^9^. Uma compreensão abrangente do CET é fundamental para garantir o diagnóstico precoce e o acompanhamento e tratamento médicos adequados. Esta revisão abrange aspectos genéticos, características clínicas da doença com foco nas manifestações renais e aborda aspectos-chave do diagnóstico e tratamento das lesões renais associadas ao CET.

## Aspectos Genéticos

O CET é causado por variantes patogênicas nos genes *TSC1* e *TSC2*, reconhecidos como genes supressores de tumores, localizados nos cromossomos 9q34.13 e 16p13.31, respectivamente^10^. Esses genes são coexpressos em todas as células nucleadas e, portanto, em múltiplos órgãos, como pulmões, rins, cérebro e pâncreas. Em indivíduos saudáveis, os produtos gênicos tuberina e hamartina formam heterodímeros com alta afinidade, constituindo um complexo que regula negativamente a cascata do *alvo mecanístico da rapamicina (mTOR)*. Essa cascata desempenha um papel fundamental no crescimento e proliferação celular por meio da biossíntese ribossômica e da síntese de proteínas^10^. Pacientes com CET apresentam variantes patogênicas em *TSC1* ou *TSC2*, mas não em ambos os genes; essas variantes patogênicas inativam funcionalmente *TSC1* ou *TSC2* ou levam à perda da conformação ideal do complexo hamartina-tuberina, causando ativação aberrante da via mTOR e resultando em aumento da proliferação e crescimento celular (Figura 1)^4,10,11^. Embora a perda de um único alelo de *TSC1* ou *TSC2* possa ser suficiente para induzir determinadas características clínicas do CET, como alterações neuropsiquiátricas, o desenvolvimento de hamartomas parece exigir uma mutação somática inibitória adicional, denominada segundo golpe, no alelo remanescente. Isso se alinha com a clássica hipótese dos dois golpes de Knudson^4,10,12^. Os segundos golpes são frequentemente identificados no alelo remanescente do *TSC1* ou *TSC2* na maioria dos carcinomas de células renais e AML relacionados ao CET^13,14^.

**Figura 1:** Disfunção da via mTOR e mecanismo de ação dos inibidores de mTOR no complexo da esclerose tuberosa.

Existe uma gama diversificada de variantes patogênicas em ambos os genes. As variantes patogênicas no gene *TSC1* são frequentemente pequenas inserções ou deleções que resultam em proteínas mais curtas. As variantes patogênicas no gene *TSC2* incluem grandes deleções, mutações *nonsense* (quando uma mutação pontual em uma sequência de DNA resulta em um códon de parada prematuro que causa a cessação da tradução e impede a síntese de uma proteína completa) e mutações *missense* (quando um único nucleotídeo muda resultando em um aminoácido diferente)^10^. De acordo com o *Leiden Open Variation Database*, mais de 900 variantes alélicas patogênicas no *TSC1* e 2700 no *TSC2* foram relatadas até o momento^15^. Notavelmente, as variantes patogênicas no *TSC2* são mais prevalentes do que no *TSC1*, com variantes patogênicas *missense* e deleções genômicas significativas sendo mais comumente observadas no *TSC2* ^10^. Variantes patogênicas *de novo* constituem aproximadamente 80% dos casos de CET, sendo aproximadamente quatro vezes mais comuns no *TSC2* do que no *TSC1*. Em casos familiares, não há diferença entre a prevalência de variantes patogênicas no *TSC1* e *TSC2* ^10,13^. Apesar da ausência de uma clara correlação genótipo-fenótipo, as variantes patogênicas em *TSC2* frequentemente estão associadas a manifestações clínicas mais graves de angiomiolipoma renal, comprometimento cognitivo e epilepsia^10^.

## Manifestações Clínicas e Critérios Diagnósticos

Existe um amplo espectro de fenótipos em termos de idade de início, manifestações clínicas, gravidade e número de lesões^5,16^. Aproximadamente 90% dos pacientes apresentam lesões cutâneas, 90% apresentam algum sinal ou sintoma neurológico e 75–80% apresentam anormalidades renais^16^.

As manifestações clínicas têm características distintas em termos de início dos sintomas. Os rabdomiomas cardíacos podem ser detectados durante a vida intrauterina em 90% dos indivíduos afetados e frequentemente regridem na primeira infância. Os bebês frequentemente desenvolvem uma forma específica de epilepsia caracterizada por espasmos, astrocitomas subependimários de células gigantes (ACG), epilepsia, dificuldades cognitivas e distúrbios neuropsiquiátricos, coletivamente denominados distúrbios neuropsiquiátricos associados ao CET (TAND, por sua sigla em inglês), que são reconhecíveis na primeira infância. As manifestações dermatológicas geralmente são diagnosticadas na infância, as manifestações renais podem começar durante a infância e persistir até a idade adulta, enquanto a LAM ocorre principalmente em mulheres a partir da adolescência, possivelmente associada a fatores hormonais^5,7,16^ (Figura 2).

**Figura 2:** Manifestações clínicas do Complexo Esclerose Tuberosa.

O diagnóstico de CET pode ser estabelecido por meio de genotipagem e/ou manifestações clínicas^17^. A identificação de uma variante patogênica no *TSC1* ou *TSC2* é suficiente para o diagnóstico de CET, independentemente dos achados clínicos, uma vez que as manifestações de CET podem surgir em diferentes idades^17^. No entanto, em 10 a 15% dos pacientes com CET que preenchem os critérios de diagnóstico clínico, a variante patogênica pode escapar à identificação por meio de testes genéticos convencionais. Isso pode ser atribuído à presença de mosaicismo e mutações intrônicas, indicando que a ausência de uma variante patogênica identificada não exclui o diagnóstico de CET^14^.

Os critérios clínicos para o diagnóstico de CET foram revisados recentemente^17^. Um diagnóstico clínico definitivo requer a presença de dois critérios maiores ou um critério maior e dois menores. A presença de um critério maior ou dois critérios menores sugere um possível diagnóstico de CET (Quadro 1)^17^.

**Quadro 1:** Critérios diagnósticos para o complexo da esclerose tuberosa (CET)

**Critérios genéticos** * Presença de uma variante patogênica no *TSC1* ou *TSC2*	
**Critérios clínicos** Necessário dois critérios maiores ou um critério maior e dois critérios menores	
**Critérios maiores**	**Critérios menores**
Angiofibromas (≥ 3) ou placa cefálica fibrosa	Corrosão do esmalte dentário (≥ 3)
Fibromas ungueais (≥ 2)	Fibromas intraorais (≥ 2)
Máculas hipomelanóticas (≥ 3, com pelo menos 5 mm de diâmetro)	Hamartoma não renal
Mancha de Shagreen	Mancha acrômica na retina
Múltiplos hamartomas na retina	Múltiplos cistos renais
Múltiplos tubérculos corticais e/ou linhas de migração radial	Lesões cutâneas do tipo “confete”
Nódulos subependimários (≥ 2)	Lesões ósseas escleróticas
Astrocitomas de células gigantes subependimais	
Angiomiolipomas renais (≥ 2)**	
Rabdomioma cardíaco	
Linfangioleiomiomatose**	

* *Identificação de uma mutação genética claramente patogênica que impede a síntese proteica e/ou inativa a função das proteínas TSC1 ou TSC2; outras variantes devem ser avaliadas com cautela.*

** *A combinação de angiomiolipomas e linfagioleiomiomatose sem outros achados não preenche os critérios diagnósticos.*

## Manifestações Renais Relacionadas ao CET

O envolvimento renal no CET pode ter início na primeira infância e permanecer assintomático ou oligossintomático por vários anos^18^. Sua incidência e gravidade aumentam ao longo da vida, constituindo uma das principais causas de morbidade e mortalidade^6,18^. Aproximadamente 80% das crianças apresentarão alguma manifestação renal até os 10 anos de idade, com progressão das lesões ao longo da vida^6,18^. A manifestação renal mais prevalente é o angiomiolipoma, seguido por cistos renais (Figura 3). Outros tumores renais, como o carcinoma de células renais (CCR) e oncocitoma, podem ocorrer com menos frequência^18,19^.

**Figura 3:** RM de um paciente do sexo masculino com complexo da esclerose tuberosa mostrando um grande angiomiolipoma no rim direito e um cisto no rim esquerdo.

### Angiomiolipoma Renal

O angiomiolipoma está presente em mais da metade dos pacientes com CET e em até 85% dos pacientes com lesões renais. O início pode ser precoce, afetando até 20% das crianças com menos de 2 anos de idade^6,18,19^. A condição é caracterizada por contribuições variadas de adipócitos, células musculares lisas e células endoteliais, e a análise genética revelou que o segundo golpe genético está presente em todas as três linhas celulares no angiomiolipoma, indicando que os três elementos surgem de uma célula precursora comum que sofreu inativação de ambos os alelos, *TSC1* ou *TSC2* ^4,6,10^. Acredita-se que a célula de origem do angiomiolipoma seja derivada dos pericitos renais, que são células epiteliais perivasculares com alta capacidade de diferenciação, angiogênese e acúmulo de lipídios^19^. Devido ao potencial significativo de crescimento e vascularização anormal, os angiomiolipomas podem desenvolver aneurismas intratumorais, que são propensos a rupturas espontâneas e hemorragias, principalmente em lesões com mais de 3 cm de diâmetro^6,16^. As complicações renais têm sido consideradas uma das principais causas de óbito nesses pacientes^6^.

A destruição progressiva do parênquima renal causada pelo crescimento do angiomiolipoma parece desempenhar um papel fundamental na perda da função renal (Figura 4)^6,16^. Outros fatores, como hiperfiltração glomerular compensatória, hipertensão arterial, embolizações repetidas, nefrectomia e uso de medicamentos nefrotóxicos, também podem contribuir para essa perda^4,20^. Aproximadamente 40% dos pacientes com CET apresentam uma redução prematura na taxa de filtração glomerular (TFG). Aos 50 anos de idade, eles podem apresentar doença renal crônica (DRC) em estágio 3 ou inferior, em comparação com 3% da população em geral^6^. Embora o risco de progressão para DRC em estágio 5 dialítico (DRC-5D) seja pequeno (1 a 4% dos pacientes evoluem para terapia renal substitutiva), uma doença cardiovascular acelerada relacionada à DRC representa uma ameaça significativa para esses pacientes^4,6,17^.

**Figura 4:** RM mostrando grandes massas renais compatíveis com angiomiolipomas que substituem praticamente todo o parênquima renal em um paciente com complexo da esclerose tuberosa.

### Doença Renal Cística

Os cistos renais são a segunda manifestação renal mais frequente associada ao CET^6,17^. Podem surgir já na infância e são considerados a segunda lesão renal mais comum, afetando até metade de todos os pacientes no decorrer da vida^18^. Os cistos renais podem ser únicos ou múltiplos e geralmente são assintomáticos. No entanto, eles são suscetíveis a complicações conforme observado em outras doenças renais císticas, como hemorragia, infecção e dor^19,20^. Em até 2–3% dos pacientes, grandes deleções no *TSC2* podem afetar o gene adjacente *PKD1*, ambos localizados no cromossomo 16, resultando na síndrome de deleção dos genes contíguos *PKD1/TSC2*, caracterizada por doença cística grave, de início precoce e acelerada. Os indivíduos com essa síndrome apresentam múltiplos cistos renais identificáveis ao nascimento, alterações na concentração urinária, hipertensão e rápida perda da função renal, que pode progredir para DRC-5D na adolescência^6,19^.

O mecanismo de formação de cistos renais relacionados ao CET ainda não é totalmente compreendido. Diferentemente do angiomiolipoma, não há perda de heterozigosidade devido a um segundo golpe, e a expressão de tuberina e hamartina foi observada nos cistos^21,22^. Estudos experimentais utilizando um modelo de camundongo com o gene TSC2 inibido sugerem que as vesículas extracelulares desempenham um papel importante na cistogênese relacionada ao CET^23–25^.

### Carcinoma de Células Renais e Oncocitoma

A incidência de carcinoma de células renais (CCR) no CET é 2 a 4 vezes maior (2–4% vs. 1%) e aparece mais cedo no decorrer da vida (~30 vs. ~55 anos) do que na população em geral^19^. Os CCRs geralmente são múltiplos e bilaterais, com crescimento mais rápido que o do angiomiolipoma e, normalmente, sem conteúdo lipídico^26,27^. Existe uma predominância entre mulheres, o que contrasta mais uma vez com a população em geral, onde o CCR é mais comum entre os homens^26,27^. Vários tipos histológicos de CCR foram descritos, incluindo estroma fibromiomatoso, CCR eosinofílico sólido e cístico, eosinofílico vacuolar e CCR oncocítico de baixo grau^28^.

Por fim, o oncocitoma é um tumor benigno raro, geralmente unilateral e solitário, com crescimento rápido e grande, mas sem comportamento invasivo. O diagnóstico histológico de oncocitoma baseia-se na identificação de células granulares intensamente eosinofílicas. A ressecção cirúrgica continua sendo o tratamento de escolha^19^.

## Inibidores de mTOR: Uma Terapia Específica para CET

Avanços na compreensão da fisiopatologia do CET abriram caminho para a utilização de inibidores de mTOR, ou seja, sirolimus e everolimus, para o tratamento dessa doença. Do ponto de vista renal, os inibidores de mTOR promovem o controle do crescimento e dificultam a progressão do angiomiolipoma, reduzindo a necessidade de intervenções cirúrgicas e invasivas, como nefrectomia e embolizações repetidas, que levam à perda de massa e função renal^4,17,19^.

Estudos experimentais em um modelo animal de CET demonstraram que o tratamento com sirolimus inativou de forma eficaz a via mTOR, resultando em uma redução substancial de tumores renais, maior sobrevida e melhor estado clínico^29^. O impacto do sirolimus na resposta tumoral tem sido associado à indução de apoptose, diminuição do tamanho celular e necrose, possivelmente devido aos seus efeitos tumorais pró-trombóticos ou antiangiogênicos^23,25,30^.

A evidência clínica inicial que apoia os benefícios potenciais do sirolimus em pacientes com CET foi relatada em um paciente de 19 anos em 2006^31^. Posteriormente, um estudo clínico aberto e não randomizado avaliou o efeito do sirolimus na redução do volume do angiomiolipoma em pacientes com CET ou LAM esporádica^32^. Dos 25 pacientes incluídos, 20 completaram o acompanhamento de 24 meses, abrangendo 12 meses iniciais de tratamento (dose inicial de 0,25 mg/m^2^ com ajustes para manter os níveis séricos em 10–15 ng/mL) seguidos de 12 meses sem medicação. Uma redução média de aproximadamente 50% no tamanho basal do angiomiolipoma e uma tendência ao aumento do volume após a interrupção da medicação foram observadas pela RM^32^. Estudos subsequentes que avaliaram o sirolimus demonstraram consistentemente sua eficiência no controle do angiomiolipoma renal. Em um estudo aberto, multicêntrico, não randomizado, de fase 2, que avaliou 16 pacientes com CET ou LAM esporádica e angiomiolipoma renal em uso de sirolimus por até 24 meses (nível sérico de 3–10 ng/mL), observou-se uma redução sustentada no diâmetro do angiomiolipoma em todos os pacientes, com a maior redução no primeiro ano de terapia^33^. Outros estudos clínicos de fase 2 relataram efeitos semelhantes, acompanhados por uma diminuição nos níveis séricos do fator de crescimento do endotélio vascular D (VEGF-D)^34–38^. Mais recentemente, em um estudo retrospectivo, Watanabe et al. analisaram os exames de tomografia computadorizada de 14 pacientes com CET em uso de sirolimus e demonstraram que a diminuição do tamanho do angiomiolipoma ocorre principalmente devido à redução dos compartimentos hipervascularizados e pobres em gordura^38^. Eles também relataram uma redução acentuada no diâmetro de aneurismas e em ectasias vasculares intratumorais^38^. É importante mencionar que pacientes em uso de sirolimus requerem monitoramento rigoroso, pois podem apresentar eventos adversos como estomatite, lesões cutâneas, dislipidemia e infecção respiratória, entre outros, cuja frequência e gravidade geralmente melhoram com o passar do tempo^32,33,39–42^.

O everolimus, um análogo do sirolimus com maior disponibilidade oral e menor ligação proteica, foi investigado para o tratamento do CET no estudo EXIST-1, que analisou seus efeitos na redução dos ACGs^40^. Posteriormente, um estudo de fase 3 multicêntrico, randomizado, duplo-cego e controlado por placebo (EXIST-2) avaliou a resposta do angiomiolipoma renal ao everolimus (10 mg diários) em pacientes adultos (N = 118) com CET ou LAM esporádica com angiomiolipoma ≥ 3 cm de diâmetro^41^. A exposição média foi de 38 semanas para o grupo everolimus e 34 semanas para o grupo placebo. A taxa de resposta na redução do AML, ou seja, redução de pelo menos 50% do volume total do angiomiolipoma, foi de 42% para o grupo everolimus [33 de 79 (IC 95% 31–53%)] e 0% para o placebo, com um tempo mediano de resposta ao everolimus de 2 a 9 meses^41^. O estudo de extensão EXIST-2 demonstrou que o efeito do tratamento se mantém ao longo do tempo, com um aumento na taxa de resposta em comparação com o estudo primário, de 42% para 54%. Além disso, aproximadamente 97% dos pacientes apresentaram uma redução no angiomiolipoma em comparação com o valor basal^42^. Nenhum dos pacientes em uso de everolimus apresentou sangramento renal^42^. Ademais, a análise *post hoc* de pacientes pediátricos (N = 33) do estudo EXIST-1 demonstrou a segurança e a eficácia do everolimus no tratamento de angiomiolipoma renal nessa população^43^. É importante ressaltar que a TFGe média dos pacientes dos estudos EXIST-1 (N = 111) e EXIST-2 (N = 112) que usaram everolimus permaneceu estável durante todo o período de acompanhamento. Um declínio na TFGe foi observado somente em alguns pacientes que já apresentavam alterações significativas na função renal pré-tratamento. Ainda, a presença de proteinúria, avaliada por meio de exame de urina com tira reagente após o início do everolimus, foi considerada leve na maioria dos casos^44^. Dados do mundo real confirmaram a eficácia e segurança do uso de everolimus em uma dose média diária de 8,4 mg para o tratamento de angiomiolipoma renal relacionado ao CET, promovendo regressão e estabilização das lesões por um período de até 3 anos^45^. Recentemente, a atualização de uma revisão sistemática Cochrane, que incluiu dados de pacientes (N = 703) de seis ensaios clínicos randomizados, confirmou que o everolimus pode reduzir o tamanho do angiomiolipoma em 50% (risco relativo 24,69; IC 95% 3,51 a 173,41; P = 0,001), além de reduzir o tamanho do ACG e a frequência de convulsões e melhorar as lesões cutâneas^46^. Embora não tenha sido observada nenhuma diferença no número total de efeitos adversos entre os grupos tratamento e placebo, mais participantes do primeiro grupo precisaram reduzir a dose, interromper ou descontinuar a medicação e apresentaram efeitos adversos mais graves^46^.

Estudos têm relatado de forma consistente que os inibidores de mTOR estão associados ao controle do crescimento do angiomiolipoma, reduzindo tanto seu tamanho quanto o risco de sangramento e a necessidade de intervenção cirúrgica, ao mesmo tempo em que preservam a função renal com uma incidência relativamente baixa de efeitos adversos graves^32–48^. Uma vez que os inibidores de mTOR, por terem apenas efeitos citostáticos, devem ser usados indefinidamente, talvez por toda a vida, para garantir seus efeitos benéficos, é importante estar ciente de seus efeitos adversos (Quadro 2) e elaborar estratégias para identificá-los e manejá-los adequadamente. O monitoramento laboratorial e clínico regular dos possíveis efeitos adversos, a educação dos pacientes e, sempre que necessário, a redução da dose ou a suspensão temporária da medicação são partes essenciais do cuidado com o paciente^49^. Além disso, com o objetivo de reduzir a ocorrência de efeitos colaterais, novos protocolos para o uso de inibidores de mTOR no tratamento do CET têm sido avaliados. Um recente estudo prospectivo comparou o uso de everolimus em dose padrão (N = 23; 10 mg/dia por 12 meses) com dosagem sequencial (N = 30; 10 mg/dia por 4 meses, seguido de 5 mg/dia até o 12º mês)^50^. O grupo de tratamento sequencial apresentou eficácia semelhante à dose padrão, com menor incidência de efeitos adversos e menor custo^50^. Adicionalmente, em um estudo prospectivo com acompanhamento de 48 meses (N = 11), no qual o everolimus foi iniciado em doses mais baixas de 2,5 mg/dia e aumentado para 5 mg/dia de acordo com a tolerância e o nível sérico (8–15 ng/mL), a massa tumoral do angiomiolipoma diminuiu a partir do sexto mês de tratamento e permaneceu estável durante todo o estudo^51^.

**Quadro 2:** Principais efeitos adversos relacionados ao uso dos inibidores de mTOR sirolimus e everolimus

	Everolimus	Sirolimus
**Infeccioso**	Trato respiratório superior, infecção do trato urinário, pneumonia	Trato respiratório superior, pneumonia, celulite, infecção urinária
**Hematológico**	Leucopenia, anemia	Leucopenia, anemia
**Metabólico**	Dislipidemia, hipofosfatemia	Dislipidemia, hipocalemia
**Neurológico**	Cefaleia, convulsão	Cefaleia, tontura, tremor
**Gastrointestinal**	Estomatite, dor abdominal, náusea, vômito	Estomatite, diarreia, náusea e dor abdominal
**Dermatológico**	Acne, eczema	Acne, foliculite
**Ginecológico**	Amenorreia, irregularidade menstrual	Amenorreia, irregularidade menstrual
**Geral**	Artralgia, fadiga	Edema periférico, fadiga
**Laboratorial**	Aumento na LDH	Aumento de LDH, TGO e TGP
**Renal**	Proteinúria	Proteinúria
**Cardíaco**		Taquicardia, pressão arterial elevada

* *LDH: enzima lactato desidrogenase; TGO: transaminase glutâmico-oxalacética; TGP: transaminase glutâmico-pirúvica.*

Outra questão importante sobre o uso de inibidores de mTOR no CET que merece nossa atenção é se um dos medicamentos comercialmente disponíveis, sirolimus ou everolimus, é superior ao outro. Como possuem uma estrutura molecular semelhante, é razoável supor que ambos possam proporcionar benefícios similares aos pacientes com CET^52^. Infelizmente, a falta de estudos clínicos randomizados que avaliem o sirolimus para o tratamento do angiomiolipoma renal relacionado ao CET tornou-se um obstáculo à aprovação desse medicamento pelas agências reguladoras para essa finalidade. Recentemente, um estudo retrospectivo multicêntrico sugeriu que ambos os medicamentos são boas opções terapêuticas, com o everolimus parecendo ser ligeiramente superior na redução do angiomiolipoma renal relacionado ao CET^53^. O everolimus foi aprovado pela *Food and Drug Administration* (FDA) e pela *European Medicines Agency* (EMA) para o tratamento de angiomiolipoma renal ≥ 3 cm de diâmetro em pacientes adultos com CET em 2016 e 2018, respectivamente^54,55^. Atualmente, é considerada a terapia de primeira linha para o tratamento eletivo de angiomiolipoma^17^. No Brasil, o sirolimus foi incorporado ao sistema nacional de saúde brasileiro para o tratamento da LAM (Portaria SCTIE/MS nº 24, de 4 de agosto de 2020) (Figura 5)^56^. A Agência Nacional de Vigilância Sanitária (ANVISA) atualmente autoriza a comercialização e o uso de everolimus para o tratamento de angiomiolipoma associado ao CET^57^.

**Figura 5:** RM de uma paciente do sexo feminino com complexo da esclerose tuberosa e linfangioleiomiomatose. Observa-se pneumotórax, uma complicação da linfangioleiomiomatose, com atelectasia pulmonar ipsilateral.

Em contraste com os dados convincentes sobre a eficácia dos inibidores de mTOR em pacientes com angiomiolipoma, a eficácia desses agentes no tratamento da doença renal cística associada ao CET ainda não foi totalmente definida, mas eles têm apresentado resultados promissores em pesquisas recentes^20,58^. Outro aspecto importante é que, embora o everolimus tenha sido utilizado como opção de tratamento para o CCR avançado na população em geral^59,60^, a eficácia dos inibidores de mTOR para CCR no CET permanece incerta^61–64^.

## Cuidados Nefrológicos e Acompanhamento no CET

Pacientes com CET precisam de acompanhamento nefrológico como parte integrante do cuidado multidisciplinar desde a infância até a idade adulta para garantir o diagnóstico precoce de alterações renais, o monitoramento periódico da função renal e o início de um tratamento específico quando indicado. De acordo com o mais recente consenso internacional sobre o CET, recomenda-se a realização de exames de imagem, de preferência RM, para avaliar lesões renais, como angiomiolipoma e cistos, em pacientes recém-diagnosticados ou suspeitos. Idealmente, a RM deve ser repetida a cada 1 a 3 anos para avaliar o crescimento e a progressão da lesão, independentemente da modalidade terapêutica^17^. Distinguir CCRs de angiomiolipomas, especialmente aqueles com baixo teor de gordura, pode ser um desafio^19^. Embora diversas técnicas ajudem nessa diferenciação, o rápido crescimento de lesões pré-existentes e as alterações na morfologia do cisto sugerem potencial malignidade. A RM destaca-se como o método de imagem padrão-ouro para diagnóstico^20^. Em casos onde a RM é inconclusiva, pode ser necessária uma biópsia^17,19^.

A taxa de filtração glomerular, estimada a partir da creatinina sérica e/ou cistatina C, e a proteinúria devem ser monitoradas ao menos uma vez por ano^17^. A hipertensão é mais comum em pacientes com CET do que na população em geral. Portanto, o controle da pressão arterial é essencial e pode ajudar a retardar a progressão da DRC^6^. Inibidores da enzima conversora de angiotensina e bloqueadores do receptor de angiotensina são os medicamentos preferidos para esse fim, já que podem ter um efeito supressor no crescimento tumoral na AML e nos cistos^6^.

Para terapia específica, os inibidores de mTOR são o tratamento de primeira linha para pacientes assintomáticos com angiomiolipomas > 3 cm de diâmetro^17^. Doses de everolimus inferiores às recomendadas pelo estudo EXIST-2, como 5 mg/dia, têm demonstrado boas respostas terapêuticas^17,51^. Embolização seletiva e nefrectomia parcial são possíveis terapias de segunda linha para angiomiolipoma assintomático com mais de 3 cm de diâmetro^17^. Nos casos de AML com hemorragia aguda, indica-se a embolização arterial, seguida de corticoterapia para prevenir a síndrome pós-embolização^17^.

Em termos de terapia renal substitutiva, todas as modalidades terapêuticas podem ser consideradas^17^. O transplante renal é uma opção viável, apresentando taxas de sobrevida do enxerto mais elevadas do que na população em geral^65^. Vale ressaltar que os inibidores de mTOR podem ser benéficos na prevenção do sangramento tumoral, mesmo na DRC avançada. Portanto, seu uso deve ser contemplado em pacientes com CET sob terapia dialítica e incluído no regime imunossupressor para transplante renal^66–68^. Por fim, o tratamento do CCR é adaptado ao estadiamento do tumor e abrange diversas modalidades terapêuticas, como radioterapia, imunoterapia e/ou ressecção cirúrgica^69^. A Quadro 3 resume as principais recomendações para o manejo da LMA relacionada ao CET.

**Quadro 3:** Principais recomendações para o cuidado nefrológico de pacientes com CET

**Diagnóstico e acompanhamento**	
Controle da pressão arterial	Realizar acompanhamento de acordo com as diretrizes atuais desde o diagnóstico de CET. Dar preferência ao uso de IECA/BRA.
TFGe	Ao menos uma vez por ano
Proteinúria	Ao menos uma vez por ano
Exame de imagem (preferencialmente RM)	Realizar quando houver diagnóstico ou suspeita de CET para auxiliar na confirmação do diagnóstico. Repetir a cada 1 a 3 anos para avaliar o crescimento e a progressão das lesões.
**Manejo de angiomiolipomas**	
Pacientes assintomáticos com angiomiolipomas < 3 cm de diâmetro	Acompanhamento
Pacientes assintomáticos, > 18 anos de idade, com angiomiolipomas > 3 cm de diâmetro	Terapia de primeira linha: inibidores de mTOR. Monitorar a dose de acordo com efeitos adversos e nível sérico. Opções terapêuticas: nefrectomia parcial e embolização arterial seletiva
Angiomiolipomas com sangramento agudo	Terapia de primeira linha: embolização arterial seguida de corticoterapia sistêmica por 7 dias (para evitar síndrome pós-embolização). Se possível, evitar a nefrectomia.
**Terapia renal substitutiva no CET**	
Modalidade de TRS	Todas as modalidades podem ser utilizadas. Considere manter o uso de inibidores de mTOR para pacientes em diálise a fim de prevenir sangramento de angiomiolipomas em rins nativos e, se necessário, controlar outras manifestações da doença.
Transplante renal	Considerar o uso de inibidores de mTOR no regime imunossupressor, também para prevenir sangramento de angiomiolipomas em rins nativos e, se necessário, para controlar outras manifestações da doença. Considerar nefrectomia de rins nativos, dependendo do tamanho do rim e dos angiomiolipomas, devido ao risco de sangramento.

* *CET: complexo da esclerose tuberosa; IECA: inibidores da enzima conversora de angiotensina; BRA: bloqueadores do receptor de angiotensina; TFGe: taxa de filtração glomerular estimada; TRS: terapia renal substitutiva.*

## Perspectivas Futuras

A identificação do envolvimento da via mTOR na fisiopatologia do angiomiolipoma relacionado ao CET aprimorou nossa compreensão da doença. No entanto, as opções terapêuticas permanecem limitadas, exercendo principalmente um efeito citostático. São necessárias mais pesquisas para identificar biomarcadores, novos alvos terapêuticos e alternativas citotóxicas para o controle eficaz da doença. Nesse sentido, um recente ensaio clínico randomizado, duplo-cego e controlado por placebo investigou a metformina como uma possível opção terapêutica para tumores relacionados ao CET, devido ao seu mecanismo inibitório da via mTOR por meio da ativação da proteína quinase ativada por adenosina monofosfato (AMPK, por sua sigla em inglês) e p53. Embora tenha apresentado resultados promissores na redução do volume do ACG, não se observou efeito semelhante com o angiomiolipoma renal^70^.

Estudos *in vitro* e modelos animais sugerem possíveis alvos terapêuticos. Observou-se a influência da via mTOR no metabolismo da vitamina A e na expressão do receptor beta do ácido retinoico (RARβ)^71^. Um estudo *in vitro* usando linhas celulares com variantes patogênicas no CET mostrou menor expressão de RARβ. O ácido retinoico, um metabólito da vitamina A já utilizado no tratamento de alguns tipos de leucemia, foi capaz de normalizar os níveis de RARβ e limitar a migração celular. Embora não tenha havido efeito significativo na proliferação celular, o estudo sugere que a combinação de inibidores de mTOR com ácido retinoico pode ser uma opção terapêutica para reduzir as doses e os efeitos colaterais desses medicamentos^71^. Estudos que avaliam o efeito potencial dos inibidores de tirosina quinase, imatinib e nilotinib, no CET têm demonstrado um efeito citotóxico desses medicamentos tanto em linhas celulares de angiomiolipoma quanto de LAM, bem como uma redução no crescimento tumoral em modelos animais tratados com imatinib^72^.

A terapia gênica parece ser um campo promissor. Um estudo pré-clínico com modelos animais afetados por mutações no *TSC1* e lesões no sistema nervoso central mostrou aumento da sobrevida e restauração das funções proteicas após a administração intravenosa de um vetor viral de adenovírus que codifica a hamartina^73^. Além disso, também foi demonstrado que o uso de um vetor viral de adenovírus codificado para tuberina em um modelo animal afetado pelo *TSC2* melhorou a sobrevida e reduziu o envolvimento cerebral^74^.

Por último, mas não menos importante, regulação e função mitocondrial das linhas celulares afetadas^75^, ativação do p53 e regulação da apoptose^76^, mediadores inflamatórios do microambiente celular^77^, envolvimento do RNA circular na tumorigênese^78^, e interação alterada entre as proteínas do gene *TSC2* e as proteínas de ligação à lipoproteína de alta densidade^79^ foram considerados alvos terapêuticos em potencial no angiomiolipoma relacionado ao CET.

## Conclusões

A alta prevalência de envolvimento renal no CET, aliada à sua associação com elevada morbimortalidade, ressalta a importância de oferecer cuidados nefrológicos a esses pacientes desde a infância. A compreensão da fisiopatologia do CET foi crucial para o uso de inibidores de mTOR no tratamento desse distúrbio, abrindo caminhos para a identificação de novos alvos terapêuticos e medicamentos com maior eficácia e menos efeitos adversos para modificar o curso da doença. Pesquisas atuais e futuras com o objetivo de prevenir o início e a progressão de manifestações relacionadas ao CET, incluindo lesões renais, têm proporcionado esperança de uma vida melhor para as pessoas afetadas pelo CET e seus familiares.
